# An Autonomous Land Vehicle Navigation System Based on a Wheel-Mounted IMU

**DOI:** 10.3390/s26010328

**Published:** 2026-01-04

**Authors:** Shuang Du, Wei Sun, Xin Wang, Yuyang Zhang, Yongxin Zhang, Qihang Li

**Affiliations:** 1 School of Aeronautics and Astronautics, University of Electronics Science and Technology of China, Chengdu 611731, China; 202422100432@std.uestc.edu.cn (X.W.); zyy2tk@std.uestc.edu.cn (Y.Z.); 2 Aircraft Swarm Intelligent Sensing and Cooperative Control Key Laboratory of Sichuan Province, Chengdu 611731, China; 3 School of Geomatics, Liaoning Technical University, Fuxin 123000, China; sunwei@lntu.edu.cn; 4 Huantian Wisdom Technology Co., Ltd., Meishan 620564, China; zhangyx@htwisdom.cn (Y.Z.); liqh@htwisdom.cn (Q.L.)

**Keywords:** MEMS IMU, wheeled INS, vehicle motion constraints, system observability, extended particle filter

## Abstract

Navigation errors due to drifting in inertial systems using low-cost sensors are some of the main challenges for land vehicle navigation in Global Navigation Satellite System (GNSS)-denied environments. In this paper, we propose an autonomous navigation strategy with a wheel-mounted microelectromechanical system (MEMS) inertial measurement unit (IMU), referred to as the wheeled inertial navigation system (INS), to effectively suppress drifted navigation errors. The position, velocity, and attitude (PVA) of the vehicle are predicted through the inertial mechanization algorithm, while gyro outputs are utilized to derive the vehicle’s forward velocity, which is treated as an observation with non-holonomic constraints (NHCs) to estimate the inertial navigation error states. To establish a theoretical foundation for wheeled INS error characteristics, a comprehensive system observability analysis is conducted from an analytical point of view. The wheel rotation significantly improves the observability of gyro errors perpendicular to the rotation axis, which effectively suppresses azimuth errors, horizontal velocity, and position errors. This leads to the superior navigation performance of a wheeled INS over the traditional odometer (OD)/NHC/INS. Moreover, a hybrid extended particle filter (EPF), which fuses the extended Kalman filter (EKF) and PF, is proposed to update the vehicle’s navigation states. It has the advantages of (1) dealing with the system’s non-linearity and non-Gaussian noises, and (2) simultaneously achieving both a high level of accuracy in its estimation and tolerable computational complexity. Kinematic field test results indicate that the proposed wheeled INS is able to provide an accurate navigation solution in GNSS-denied environments. When a total distance of over 26 km is traveled, the maximum position drift rate is only 0.47% and the root mean square (RMS) of the heading error is 1.13°.

## 1. Introduction

Land vehicle navigation (LVN) systems have become increasingly important with the rapid development of intelligent transportation systems in recent years. They have been widely applied in various tasks, such as unmanned driving and vehicle tracking systems [1,2,3]. The integration of GNSS and INS is widely employed in LVN systems due to the complementary features of each system, and Kalman filtering is commonly used to fuse the data from both systems [4,5,6]. Although high-end (navigation- or tactical-grade) INSs offer navigation solutions with high precision, their considerable size, price, and power consumption restrict their applications. Thanks to the significant advances in microelectromechanical technologies, the microelectromechanical system (MEMS) inertial measurement unit (IMU) is characterized by a small size, a light weight, and low costs compared to high-end inertial sensors. As a result, commercially affordable MEMS IMUs are very popular in the low-grade inertial system market and have become an attractive choice for intelligent transportation applications [7,8]. A major issue for the integrated GNSS/INS is that the GNSS signal is prone to blockage due to shelters such as buildings, tunnels, and trees in urban areas [9,10,11], during which the navigation system will drift in standalone INS mode. This is even more critical for MEMS IMUs. Their high level of noise, significant instability of bias, and extreme stochastic variance make it quite challenging to use MEMS INSs in autonomous mode for extended periods [12,13].

The odometer (OD), which measures traveling distance and velocity, is a self-contained sensor for land vehicles. Combined with non-holonomic constraints (NHC), which constrain the velocity components of the upward and transverse axes to be close to zero in land vehicles, a three-dimensional velocity has been formed to suppress the INS error due to drifting during GPS outages [14,15,16]. These odometer- and NHC-aided INSs (OD/NHC/INSs) have been proven to be an efficient solution to provide continuous navigation estimates for wheeled land vehicles. The main issue of such a system is that the observability of certain states is poor in the absence of maneuvers, which leads to accumulated position, velocity, and attitude (PVA) estimates. Moreover, data fusion from different sensors increases the complexity of hardware modification and data synchronization [17].

In recent studies, an IMU has been placed at the center of a land vehicle’s wheel to develop an autonomous navigation system. Firstly, a two-dimensional (2D) dead reckoning (DR) system is proposed based on a wheel-mounted IMU. In such a system, measurements from the accelerometer perpendicular to the wheel rotation axis are used to derive the wheel rotation angle, which can be converted into traveled distance by multiplying this figure with the wheel radius, while the vehicle azimuth is calculated from the gyro measurements [18]. The rotation of an IMU can modulate the constant sensor biases and their random drift, such as velocity or angular random walk and 1/f noise [19,20]. As a result, the impacts of these errors on navigation solutions are canceled out after a complete rotation cycle. A more complicated wheel-mounted DR system, which is similar to the OD/NHC/INS, has also been designed. The measurements from the triaxial accelerometer and gyros are used to determine the vehicle PVA solution through a mechanization algorithm. Meanwhile, the gyro data is used to determine the vehicle’s forward velocity, which is treated as an external observation with NHCs to update the vehicle navigation state [21,22]. As the IMU cannot be perfectly mounted at the land vehicle wheel’s center, the wheel rotation rate introduces significant centripetal acceleration due to the displacements between the IMU’s sensitive axes at its center and the wheel rotation center. Manual calibration is usually implemented to compensate for such detrimental effects. A calibration procedure is also proposed for the misalignment between the sensitive axes of the wheel-mounted IMU and the wheel surface [23,24]. The proposed system outperforms traditional OD/NHC/INSs in terms of navigation performance.

Significant efforts have been made to develop wheel-mounted DR systems; however, there are three main deficiencies pertaining to previous studies. Firstly, the displacements between the IMU’s sensitive axes center and wheel rotation center are calibrated manually. As the IMU’s sensitive axes center is difficult to determine, especially when the inertial chips are already packaged into a module, manual calibration is difficult to perform; therefore, online calibration of the displacements is necessary. Secondly, the system error characteristic has not been thoroughly analyzed from the perspective of observability. The reason that wheel-mounted systems are superior to traditional OD/NHC/INSs has been simply attributed to the rotation modulation of inertial sensor errors, and the fundamental reasons have not yet been understood. Thirdly, in the existing literature, the data fusion of vehicle constraints and inertial data was simply implemented using an extended Kalman filter (EKF); however, bumpy road conditions and vehicle maneuvers may increase the complexity of non-linearity and non-Gaussian noise in the system, disrupting the assumptions of Kalman filtering regarding the system and observation model noise.

In this paper, we propose an autonomous navigation system based on a single wheel-mounted MEMS IMU, which is referred as wheeled INS. The vehicle velocity constraints, formed by the NHC and forward velocity derived from the gyro data, are employed as observations to estimate the inertial navigation errors. To address the deficiencies in previous studies, a comprehensive observability analysis is conducted from an analytical perspective, establishing a theoretical foundation for wheeled INS error characteristics. This reveals that the improved system observability due to wheel rotations is the main reason that the wheeled INS outperforms the OD/NHC/INS in terms of navigation performance. Moreover, a hybrid filter that fuses the EKF and PF is proposed to deal with non-linearity and non-Gaussian noise in the system. A comparison of our study and the existing literature is provided in Table 1. More specifically, our findings can be summarized as follows:

1. An autonomous navigation system is proposed and implemented based on a single wheel-mounted MEMS IMU, in which the displacements between the IMU’s sensitive axes center and the IMU’s rotation center, as well as the gyro scale factor and non-orthogonal errors, is estimated online; its navigation performance is investigated through kinematic field tests, which indicate that the proposed wheeled INS is able to provide reliable PVA solutions in GNSS-denied environment.
2. The observability of wheeled INS is studied thoroughly and analytically, which identifies that the azimuth error is unobservable under vehicle normal dynamics and becomes the main cause of velocity and position errors in the east and north directions. Thanks to the improved observability of the gyro errors perpendicular to the rotation axis, the azimuth errors can be effectively suppressed. This leads to the higher navigation accuracy of wheeled INS over the OD/NHC/INS, in which both of the azimuth error and the associated gyro bias are unobservable.
3. To ensure a high level of estimation accuracy and limit the number of particles, we proposed a hybrid EKF and PF (referred to as EPF) to continuously estimate the land vehicle navigation states. The impact of the initial particle numbers on EPF estimation accuracy is also studied, and the performance of EPF is evaluated through kinematic field tests.

The remainder of the paper is organized as follows. Section 2 presents the wheeled INS navigation strategy, including the prerequisites, as well as the system and measurement mathematical models. The system observability analysis and the proposed hybrid filtering method are presented in Section 3 and Section 4, respectively. Section 5 presents the conducted field test and an analysis of the results, and finally, the conclusions of this study and future directions are summarized in Section 6.

## 2. Wheeled INS Algorithm

### 2.1. Coordinate Frames and Misalignments

**Figure 1:**
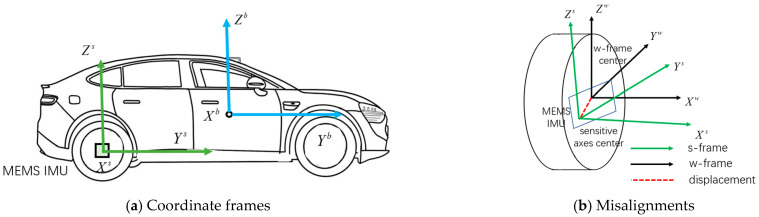
Wheel-mounted IMU, coordinate frames, and the misalignments.

In the proposed wheeled INS, the IMU is mounted at the center of the vehicle’s rear wheel, as shown in Figure 1a. The vehicle body frame (b-frame) is defined as having its origin in the vehicle mass center, with the Y- and Z -axes pointing in forward and upward directions, respectively, and the X-axis pointing in a lateral direction following the right-hand rule. The wheel frame (w-frame) is defined as having its the origin in the wheel center, with the X-axis pointing to the right of the vehicle, and the Y- and Z-axes being parallel to the wheel surface to complete the right-hand orthogonal frame. The sensor frame (s-frame) is the IMU sensitive-axis frame. Its X-axis is aligned to the rotation axis of the wheel, pointing to the right of the vehicle, to avoid singularity of the pitch angle (±90°). Based on the coordinate definitions, it can be considered that there is only a periodic roll angle difference between the w-frame and the b-frame under stable vehicle structural conditions. Moreover, the heading difference between the wheel-mounted IMU and the vehicle is approximately 90° [22]. The position, velocity, and attitude (PVA) solutions are resolved in the navigation frame (n-frame), with the origin coinciding with the b-frame, the x-axis directed towards the east, the y-axis towards the geodetic north east, and the z-axis vertically upward.

Linked to the vehicle movement, the rotation matrix between the s-frame and the b-frame is given in Equation (1).

(1) $$C_{b}^{s}=\left[\begin{array}{ccc} 1 & 0 & 0\\ 0 & \cos\omega t & \sin\omega t\\ 0 & -\sin\omega t & \cos\omega t \end{array}\right]$$

where $C_{b}^{s}$ represents the rotation matrix from the b-frame to the s-frame, and ω represents the wheel rotation rate.

In practice, the displacement between the IMU center and wheel rotation center is inevitable, as depicted in Figure 1b. The displacement would introduce centripetal acceleration to the accelerometer measurements, leading to navigation errors, especially when the vehicle drives fast. The angle misalignment between the w- and s-frames would cause erroneous projections of the vehicle angular rate in the b-frame, and also cause periodic deviations of the computed heading from the true value. Displacement can be carefully manually measured or estimated by augmenting it into the state space. Regarding the angle misalignment, we adopted a calibration method that has been proposed to solve a similar problem, namely the mounting angle errors between the pipeline inspection gauges (PIGs) body frame and the IMU body frame in the IMU-based PIGs [23,24]. As the roll misalignment angle has no impact on wheeled INS, we only take the pitch and heading misalignment angles into consideration.

### 2.2. System and Measurement Models

**Figure 2:**
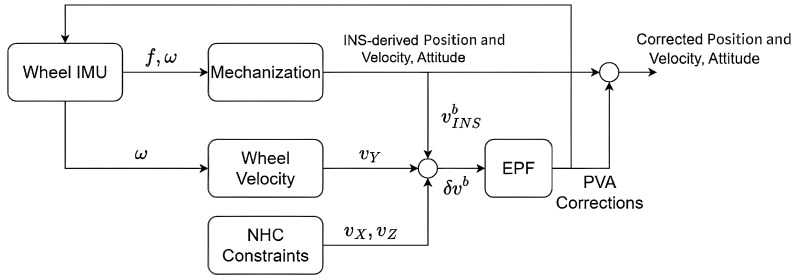
Flowchart of proposed wheeled INS.

Figure 2 illustrates the flowchart of the wheeled INS. The mechanization algorithm is employed to predict the PVA solutions based on the outputs of wheel-mounted IMU. Meanwhile, the gyro outputs and wheel radius are used to estimate the forward vehicle velocity. Combined with the NHC, they formed the body frame velocity, which is treated as the pseudo-observation to estimate the INS errors. To deal with the system nonlinearity and non-Gaussian noise, a hybrid EPF is proposed, where the proposal distribution is generated by the EKF. The closed loop scheme is adopted, so that the inertial data is compensated by the estimates of sensor errors. As the wheel rotation rate is determined by gyro outputs, the wheeled INS is more suitable for horizonal surfaces.

The 12 common error states of an inertial system, including velocity errors, attitude errors, and accelerometer and gyro biases, are considered in the wheeled INS state space. The position error states are excluded as they are weakly related to the velocity errors of the b-frame. Although the displacement between the IMU and the wheel rotation center can be carefully measured manually, their residuals still introduce detrimental centripetal accelerations, as the wheel rotation rate is significant (usually 250–500 RPM) even under normal land vehicle dynamics [25]. Therefore, the projected displacement in the Y- and Z-axes (non-rotation axes) are augmented to the state space. Similarly, the gyro scale factor of the rotation axis as well as the non-orthogonal errors between the rotation and non-rotation axes also introduce significant gyro errors. These error states must also be considered in the state space. In summary, a total of 17 error states comprise the system state space, which is given in Equation (2). By considering the additional sensor errors motivated by wheel rotation, the accelerometer and gyro error model can be described by Equations (3) and (4), respectively.

(2) $$x={\left[\begin{array}{cccccc} \delta v^{n} & \varepsilon^{n} & \gamma^{s} & d^{s} & l & \xi \end{array}\right]}^{T}$$

(3) $$\delta f^{b}=C_{s}^{b}\left(\gamma^{s}+\Delta \gamma^{s}\right)$$

(4) $$\delta \omega^{b}=C_{s}^{b}\left(d^{s}+\Delta d^{s}\right)$$

where x represents the state vector, $\delta v^{n}={\left[\begin{array}{ccc} \delta v_{E} & \delta v_{N} & \delta v_{U} \end{array}\right]}^{T}$ are the velocity errors of the n-frame; $\delta v_{E}$, $\delta v_{N}$, and $\delta v_{U}$ represent the velocity errors in the east, north, and upward directions,, respectively; $\varepsilon^{n}={\left[\begin{array}{ccc} \varepsilon_{E} & \varepsilon_{N} & \varepsilon_{U} \end{array}\right]}^{T}$ are the attitude errors of the n-frame; $\varepsilon_{E}$, $\varepsilon_{N}$, and $\varepsilon_{U}$ represent pitch, roll, and azimuth, respectively; $\gamma^{s}={\left[\begin{array}{ccc} \gamma_{X} & \gamma_{Y} & \gamma_{Z} \end{array}\right]}^{T}$ are the accelerometer biases of the s-frame; $\gamma_{X}$, $\gamma_{Y}$, and $\gamma_{Z}$ represent the accelerometer bias in the X-, Y-, and *Z*-axes, respectively; $d^{s}={\left[\begin{array}{ccc} d_{X} & d_{Y} & d_{Z} \end{array}\right]}^{T}$ is the gyro bias vector of the s-frame; $d_{X}$, $d_{Y}$, and $d_{Z}$ represent the gyro bias in the X-, Y-, and *Z*-axes, respectively; $l={\left[\begin{array}{cc} l_{Y} & l_{Z} \end{array}\right]}^{T}$ is the displacement between the IMU and wheel rotation center; $l_{Y}$ and $l_{Z}$ represent the displacement projected in the Y- and *Z*-axes, respectively; $\xi ={\left[\begin{array}{ccc} \zeta_{X} & \zeta_{XY} & \zeta_{XZ} \end{array}\right]}^{T}$ is the gyro scale factor and non-orthogonal error vector; $\zeta_{X}$, $\zeta_{XY}$ and $\zeta_{XZ}$ represent the gyro scale factor of the X-axis, and non-orthogonal errors between the X- and Y-axes, as well as the X- and Z-axes, respectively; $\delta f^{b}$ is the accelerometer error of the b-frame, $C_{s}^{b}$ is the rotation matrix from the s-frame to the s-frame, which can calculated as ${\left(C_{b}^{s}\right)}^{T}$; $\Delta \gamma^{s}$ is the motived detrimental centripetal acceleration error by wheel rotation, which can be calculated as ${\left[\begin{array}{ccc} 0 & \omega^{2}l_{Y} & \omega^{2}l_{Z} \end{array}\right]}^{T}$; $\delta \omega^{b}$ is the gyro error of the b-frame; $\Delta d^{s}$ is the motivated gyro error by scale factor and non-orthogonal error by wheel rotation, which can be calculated as ${\left[\begin{array}{ccc} \omega \zeta_{X} & \omega \zeta_{XY} & \omega \zeta_{XZ} \end{array}\right]}^{T}$.

Based on the phi-angle model [26], the differential equations of the velocity and attitude error states are derived and given in Equations (5) and (6). The accelerometer and gyro biases are modeled as a random walk process [7,27], while the displacement $l_{Y}$ and $l_{Z}$, as well as the scale factor and non-orthogonal errors, are modeled as a random constant.

(5) $$\delta {\dot{v}}^{n}=F^{n}\varepsilon^{n}+C_{b}^{n}\delta f^{b}$$

(6) $${\dot{\varepsilon }}^{n}=-C_{b}^{n}\delta \omega^{b}$$

where $F^{n}$ is the skew-symmetric matrix of $f^{n}$, which is the specific force of the n-frame; and $C_{b}^{n}$ is the transformation matrix from the b-frame to the n-frame.

Based on a perturbation analysis for $v^{b}=C_{s}^{b}C_{n}^{s}v^{n}$, and ignoring the higher-order terms, the measurement model can be described as in (7) [28].

(7) $$\delta v^{b}=C_{n}^{b}\delta v^{n}-V^{b}C_{n}^{b}\varepsilon^{n}$$

where $\delta v^{b}$ represents the velocity errors in the body frame; $C_{n}^{b}$ is the transformation matrix from the n-frame to the b-frame, which can be calculated as ${\left(C_{b}^{n}\right)}^{T}$; and $V^{b}$ is the skew-symmetric matrix of velocity vector $v^{b}$.

## 3. Observability Analysis of Wheeled INS

Performing an observability analysis for a dynamic system is essential to determine the efficiency of a filter designed to estimate system states [29]. If the system is not observable, we cannot obtain an accurate estimate of the state, even if the noise level is negligible. In other words, the observability sets a lower limit on the estimation error [30]. This section presents an observability analysis of the wheeled INS, which establishes a theoretical foundation for its error characteristics. According to [31], a state is observable as it can be determined from the observations and their time derivatives. Therefore, the system observability is analyzed through the derivatives of the velocity error of the b-frame, which are employed as the observations in wheeled INS. The differential equation for the velocity and attitude errors of the b-frame are described as follows [32]:

(8) $$\delta {\dot{v}}^{b}=\Omega_{Z}\delta v^{b}+F\varepsilon^{b}+\delta f^{b}$$

(9) $${\dot{\varepsilon }}^{b}=\Omega_{Z}\varepsilon^{b}+\delta \omega^{b}$$

where $\delta v^{b}={\left[\begin{array}{ccc} \delta v_{X} & \delta v_{Y} & \delta v_{Z} \end{array}\right]}^{T}$, $\delta v_{X}$, $\delta v_{Y}$, and $\delta v_{Z}$ represent the velocity errors in the transverse, forward, and upward axes of the b-frame, respectively; $\Omega_{Z}=\left[\begin{array}{ccc} 0 & \omega_{Z} & 0\\ -\omega_{Z} & 0 & 0\\ 0 & 0 & 0 \end{array}\right]$ represents the vehicle rotation matrix in the b-frame, and $\omega_{Z}$ is the vehicle turning rate; $\varepsilon^{b}={\left[\begin{array}{ccc} \varepsilon_{X} & \varepsilon_{Y} & \varepsilon_{Z} \end{array}\right]}^{T}$ represents the attitude errors in the b-frame, where $\varepsilon_{X}$, $\varepsilon_{Y}$, and $\varepsilon_{Z}$ represent the attitude errors in the transverse, forward, and upward axes of the b-frame; and $F=\left[\begin{array}{ccc} 0 & g & -f_{Y}\\ -g & 0 & f_{X}\\ f_{Y} & -f_{X} & 0 \end{array}\right]$, where g is the gravity, $f_{Y}$ is the acceleration in the forward axis, and $f_{X}$ is the acceleration in the lateral axis motivated by the turning motions of the vehicle.

Normally, ground vehicles mainly move in straight lines, with occasional acceleration or turning. Therefore, we first analyze the system observability under the uniform linear motion; then, the impact of acceleration and turning motion on the observability results are further investigated. To provide a comparison to the observability of wheeled INS, the observability of OD/NHC/INS is given in Appendix A.

### 3.1. Observability of Wheeled INS Under Uniform Linear Motion

As the wheel rotation rate is constant under uniform linear motion, the time derivative of the motivated centripetal accelerometer and gyro errors given by the wheel rotation, $\Delta {\dot{\gamma }}^{s}$ and $\Delta {\dot{d}}^{s}$, are null vectors. Then the derivatives of accelerometer and gyro errors of the b-frame can be calculated via Equations (10) and (11).

(10) $$\delta {\dot{f}}^{b}={\dot{C}}_{s}^{b}\gamma^{s}+{\dot{C}}_{s}^{b}\Delta \gamma^{s}+C_{s}^{b}\Delta {\dot{\gamma }}^{s}=\Omega_{bs}^{b}\delta f^{b}$$

(11) $$\delta {\dot{\omega }}^{b}={\dot{C}}_{s}^{b}d^{s}+{\dot{C}}_{s}^{b}\Delta d^{s}+C_{s}^{b}\Delta {\dot{d}}^{s}=\Omega_{bs}^{b}\delta \omega^{b}$$

where $\Omega_{bs}^{b}$ is the skew-symmetric matrix of $\omega_{bs}^{b}$, representing the wheel rotation rate vector in the b-frame.

The time derivatives of the observations can be calculated and expressed in matrix form, as shown in Equation (12), with the vehicle rotation rate matrix $\Omega_{Z}$ becoming a null matrix under linear motion.

(12) $$\left[\begin{array}{c} \delta {\dot{v}}^{b}\\ \delta {\ddot{v}}^{b}\\ \delta {\dddot{v}}^{b}\\ \vdots \\ \overset{\left(n\right)}{\delta v^{b}} \end{array}\right]=\left[\begin{array}{cccc} 0_{3\times 3} & F & I_{3\times 3} & 0_{3\times 3}\\ 0_{3\times 3} & 0_{3\times 3} & \Omega_{bs}^{b} & F\\ 0_{3\times 3} & 0_{3\times 3} & {\left(\Omega_{bs}^{b}\right)}^{2} & F\Omega_{bs}^{b}\\ \vdots & \vdots & \vdots & \vdots \\ 0_{3\times 3} & 0_{3\times 3} & {\left(\Omega_{bs}^{b}\right)}^{n-1} & F{\left(\Omega_{bs}^{b}\right)}^{n-2} \end{array}\right]\left[\begin{array}{c} \delta v^{b}\\ \varepsilon^{b}\\ \delta f^{b}\\ \delta \omega^{b} \end{array}\right]$$

where $\overset{\left(n\right)}{\delta v^{b}}$ is the $n^{th}$-order time derivative of the velocity observation.

In Equation (12), the wheel rotation rate $\Omega_{bs}^{b}$ and its exponents only appear in the third and fourth columns of the matrix, corresponding to the accelerometer errors $\delta f^{b}$ and gyro errors $\delta \omega^{b}$, indicating that the wheel rotation strongly affects the observability of these error states. As ${\left(\Omega_{bs}^{b}\right)}^{3}=-\omega^{2}\Omega_{bs}^{b}$, the fifth-order and above of the time derivatives of the observations are linearly correlated with the lower-order time derivatives. Therefore, the first–fourth-order time derivatives of observations are calculated via Equations (13)–(16) to investigate the observable states.

(13) $$\delta {\dot{v}}^{b}=F\varepsilon^{b}+\delta f^{b}=\left[\begin{array}{c} g\varepsilon_{Y}+\delta f_{X}\\ -g\varepsilon_{X}+\delta f_{Y}\\ \delta f_{Z} \end{array}\right]$$

(14) $$\delta {\ddot{v}}^{b}=\Omega_{bs}^{b}\delta f^{b}+F\delta \omega^{b}=\left[\begin{array}{c} -g\delta \omega_{Y}\\ -\omega \delta f_{Z}+g\delta \omega_{X}\\ \omega \delta f_{Y} \end{array}\right]$$

(15) $$\delta {\dddot{v}}^{b}={\left(\Omega_{bs}^{b}\right)}^{2}\delta f^{b}+F\Omega_{bs}^{b}\delta \omega^{b}=\left[\begin{array}{c} -g\omega \delta \omega_{Z}\\ -\omega^{2}\delta f_{Y}\\ -\omega^{2}\delta f_{Z} \end{array}\right]$$

(16) $$\delta {\ddddot{v}}^{b}=-\omega^{2}\Omega_{bs}^{b}\delta f^{b}+F{\left(\Omega_{bs}^{b}\right)}^{2}\delta \omega^{b}=\left[\begin{array}{c} g\omega^{2}\delta \omega_{Y}\\ \omega^{3}\delta f_{Z}\\ -\omega^{3}\delta f_{Y} \end{array}\right]$$

Based on Equations (13)–(16), the error states $\varepsilon_{X}$, $\delta f_{Y}$, $\delta f_{Z}$, $\delta \omega_{X}$, $\delta \omega_{Y}$, and $\delta \omega_{Z}$ can be directly determined by the time derivatives of the observations, as shown in Equations (17)–(22). Apparently, these error states are considered as observable. The attitude error $\varepsilon_{Y}$ is coupled with the accelerometer errors $\delta f_{X}$, and only their linear combination $g\varepsilon_{Y}+\delta f_{X}$ is observable. In addition to the directly observable velocity errors, the total number of observable states or linear combinations of states is 10. The attitude error of the Z-axis $\varepsilon_{Z}$ is unobservable during uniform linear motion.

(17) $$\varepsilon_{X}=-\frac{g\delta {\dot{v}}^{b}(2)+\delta {\ddot{v}}^{b}(1)}{g^{2}}$$

(18) $$\delta f_{Y}=\delta {\ddot{v}}^{b}(3)/\omega$$

(19) $$\delta f_{Z}={\dot{v}}^{b}(3)$$

(20) $$\delta \omega_{X}=\frac{\delta {\ddot{v}}^{b}(2)+\omega {\dot{v}}^{b}(3)}{g}$$

(21) $$\delta \omega_{Y}=-\delta {\ddot{v}}^{b}(1)/g$$

(22) $$\delta \omega_{Z}=-\delta {\dddot{v}}^{b}(1)/g\omega$$

where $\overset{\left(n\right)}{\delta v^{b}}(i)$ represent the $i^{th}$ terms of the $n^{th}$ order time derivative of observation.

### 3.2. Effects of Forward Acceleration and Turning Motions on System Observability

#### 3.2.1. Effects of Forward Accelerations

In the presence of forward accelerations, both $\Delta {\dot{\gamma }}^{s}$ and $\Delta {\dot{d}}^{s}$ are no longer null vectors and can be derived from Equations (10) and (11), as $\delta f^{b}$ and $\delta \omega^{b}$ are observable states. Then, the displacement state vector l, as well as the gyro scale factor and the non-orthogonal error vector ξ, can be calculated via Equations (23) and (24). Once l and ξ are derived, the accelerometer bias $\gamma^{s}$ and gyro bias $d^{s}$ also can be determined from $\delta f^{b}$ and $\delta \omega^{b}$ based on Equations (3) and (4).

With forward acceleration, F becomes $\left[\begin{array}{ccc} 0 & g & -f_{Y}\\ -g & 0 & 0\\ f_{Y} & 0 & 0 \end{array}\right]$, and the first element of $\delta {\dot{v}}^{b}$ can be recalculated as $-g\varepsilon_{Y}+f_{Y}\varepsilon_{Z}+\gamma_{X}$. As $f_{Y}$ is usually much lower than gravity, the attitude error $\varepsilon_{Z}$ is still hardly observable in this combination of states. As other error states are already independently observable, the forward acceleration barely affects their observability.

(23) $${\left[\begin{array}{ccc} 0 & l_{y} & l_{xz} \end{array}\right]}^{T}=\frac{r}{2\omega f_{Y}}C_{b}^{s}(\delta {\dot{f}}^{b}-\Omega_{bs}^{b}\delta f^{b})$$

(24) $${\left[\begin{array}{ccc} IE_{xy} & SF_{y} & IE_{xz} \end{array}\right]}^{T}=\frac{r}{f_{Y}}C_{b}^{s}(\delta {\dot{\omega }}^{b}-\Omega_{bs}^{b}\delta \omega^{b})$$

#### 3.2.2. Effects of Turning Motions

During the turning motion, $\Omega_{Z}$ is no longer a null matrix, and the time derivatives of observations are then calculated and expressed in the form of a matrix, as shown in Equation (25). Similarly, the wheel rotation only affects the observability of the accelerometer and gyro errors, as its exponents only appear in the third and fourth columns of the matrix. To cancel the common terms between the time derivatives of observations, we calculated the $\overset{\left(n\right)}{\delta v^{b}}-\Omega_{Z}\overset{\left(n-1\right)}{\delta v^{b}}$, as shown in Equation (26).

(25) $$\left[\begin{array}{c} \delta {\dot{v}}^{b}\\ \delta {\ddot{v}}^{b}\\ \delta {\dddot{v}}^{b}\\ \vdots \\ \overset{\left(n\right)}{\delta v^{b}} \end{array}\right]=\left[\begin{array}{cccc} \Omega_{Z} & F & I_{3\times 3} & 0_{3\times 3}\\ \Omega_{Z}^{2} & \Omega_{Z}F+F\Omega_{Z} & \Omega_{Z}+\Omega_{bs}^{b} & F\\ \Omega_{Z}^{3} & \Omega_{Z}^{2}F+\Omega_{Z}F\Omega_{Z}+F\Omega_{Z}^{2} & \Omega_{Z}^{2}+\Omega_{Z}\Omega_{bs}^{b}+{\left(\Omega_{bs}^{b}\right)}^{2} & \Omega_{Z}F+F\Omega_{Z}+F\Omega_{bs}^{b}\\ \vdots & \vdots & \vdots & \vdots \\ \Omega_{Z}^{n} & \sum_{i=0}^{n-1}\Omega_{Z}^{n-1-i}F\Omega_{Z}^{i} & \sum_{i=0}^{n-1}\Omega_{Z}^{n-1-i}{\left(\Omega_{bs}^{b}\right)}^{i} & \sum_{i=0}^{n-2}\Omega_{Z}^{n-2-i}F\Omega_{Z}^{i}+Q_{n-1}(4)\Omega_{bs}^{b} \end{array}\right]\left[\begin{array}{c} \delta v^{b}\\ \varepsilon^{b}\\ \delta f^{b}\\ \delta \omega^{b} \end{array}\right]$$

(26) $$\left[\begin{array}{c} \delta v_{2,1}\\ \delta v_{3,2}\\ \delta v_{4,3}\\ \vdots \\ \delta v_{n.n-1} \end{array}\right]=\left[\begin{array}{cccc} 0_{3\times 3} & F\Omega_{Z} & \Omega_{bs}^{b} & F\\ 0_{3\times 3} & F\Omega_{Z}^{2} & {\left(\Omega_{bs}^{b}\right)}^{2} & F\Omega_{Z}+F\Omega_{bs}^{b}\\ 0_{3\times 3} & F\Omega_{Z}^{3} & {\left(\Omega_{bs}^{b}\right)}^{3} & F\Omega_{Z}^{2}+F\Omega_{Z}\Omega_{bs}^{b}+F{\left(\Omega_{bs}^{b}\right)}^{2}\\ \vdots & \vdots & \vdots & \vdots \\ 0_{3\times 3} & F\Omega_{Z}^{n-1} & {\left(\Omega_{bs}^{b}\right)}^{n-1} & \sum_{i=0}^{n-2}F\Omega_{Z}^{n-2-i}{\left(\Omega_{bs}^{b}\right)}^{i} \end{array}\right]\left[\begin{array}{c} \delta v^{b}\\ \varepsilon^{b}\\ \delta f^{b}\\ \delta \omega^{b} \end{array}\right]$$

where $\delta v_{n,n-1}=\overset{\left(n\right)}{\delta v^{b}}-\Omega_{Z}\overset{\left(n-1\right)}{\delta v^{b}}$.

By calculating the wheel rotation matrix $\Omega_{bs}^{b}$ and its exponents, we find that the coefficients corresponding to the accelerometer bias of the X-axis are null, which means that its observability is independent of wheel rotation. Similarly, the observability of error $\varepsilon_{Z}$ is independent of vehicle rotation, as the corresponding coefficients are also null in the vehicle rotation rate matrix $\Omega_{Z}$ and its exponents. To further investigate the system observability, we calculate the $\delta {\dot{v}}^{b}-\Omega_{Z}\delta v^{b}$ and $\delta {\ddot{v}}^{b}-\Omega_{Z}\delta {\dot{v}}^{b}$ as given in Equations (27) and (28).

(27) $$\delta v_{1,0}=\left[\begin{array}{c} -g\varepsilon_{Y}+\delta f_{X}\\ g\varepsilon_{X}-f_{X}\varepsilon_{Z}+\delta f_{Y}\\ f_{X}\varepsilon_{Y}+\delta f_{Z} \end{array}\right]$$

(28) $$\delta v_{2,1}=\left[\begin{array}{c} -g(\omega_{Z}\varepsilon_{X}+\delta \omega_{Y})\\ -g(\omega_{Z}\varepsilon_{Y}-\delta \omega_{X})-f_{X}\delta \omega_{Z}-\omega \delta f_{Z}\\ f_{X}\omega_{Z}\varepsilon_{X}+f_{X}\delta \omega_{Y}+\omega \delta f_{Y} \end{array}\right]$$

According to Equation (28), the state combination $\omega_{Z}\varepsilon_{Y}-\delta \omega_{X}$ can be derived from the second element of $\delta v_{2,1}$. As the vehicle rotation rate is usually significant (usually greater than 10 °/s), the attitude error $\varepsilon_{Y}$ can be determined based on Equation (29). Then, its coupled error states, $\delta f_{X}$, can also be determined from the first element of $\delta v_{1,0}$, as shown in Equation (30). As the centripetal acceleration $f_{X}$, motivated by turning motion, is much lower than gravity [25], the attitude error state $\varepsilon_{Z}$ is still difficult to observe in the observable combination of states $g\varepsilon_{X}-f_{X}\varepsilon_{Z}+\delta f_{Y}$ in Equation (27). Finally, all error states become observable, except the error state $\varepsilon_{Z}$.

(29) $$\varepsilon_{Y}=-\frac{\delta v_{2,1}(2)+f_{X}\delta \omega_{Z}+\omega \delta f_{Z}-g\delta \omega_{X}}{g\omega_{Z}}$$

(30) $$\delta f_{X}=\delta v_{1,0}(1)+g\varepsilon_{Y}$$

The velocity and attitude errors in the n-frame can be expressed by the errors of b-frame, as shown in Equations (31) and (32). Obviously, the azimuth error $\varepsilon_{U}$ is unobservable as it is directly determined by $\varepsilon_{Z}$. The velocity errors in the east and north directions are also unobservable as they are partially related to $\varepsilon_{Z}$. Therefore, the azimuth error has become the main cause of the navigation errors in wheeled INS, and its accumulation results in a drifted horizontal position and velocity errors.

(31) $$\delta v^{n}=C_{b}^{n}\delta v^{b}-C_{b}^{n}V^{b}\varepsilon^{b}=\left[\begin{array}{c} \cos A\delta v_{X}+\sin A\delta v_{Y}+v_{Y}\varepsilon_{Z}\cos A\\ -\sin A\delta v_{X}+\cos A\delta v_{Y}-v_{Y}\varepsilon_{Z}\sin A\\ \delta v_{Z}-v_{Y}\varepsilon_{X} \end{array}\right]$$

(32) $$\varepsilon^{n}=-C_{b}^{n}\varepsilon^{b}=\left[\begin{array}{c} -\varepsilon_{X}\cos A-\varepsilon_{Y}\sin A\\ \varepsilon_{X}\sin A-\varepsilon_{Y}\cos A\\ -\varepsilon_{Z} \end{array}\right]$$

Compared to the observability of OD/NHC/INS, which is given in the Appendix A, the IMU rotation substantially improves the observability of the gyro errors of the Z-axis, as well as the accelerometer errors of the Y-axis and its coupled error state $\varepsilon_{X}$. Although the azimuth is unobservable in wheeled INS, the improved observability of the gyro errors of the Z-axis leads to the successful estimation of and compensation for such errors, which effectively mitigates the azimuth error accumulations. In contrast, the gyro error state of the Z-axis is unobservable in OD/NHC/INS, and its failed estimation leads to drifted azimuth error, leading to the velocity errors in the east and north directions, and the horizonal position errors.

## 4. Proposed EPF of Wheeled INS

Bumpy road conditions and vehicle maneuvers, such as lateral sliding, may increase the complexity of non-linearity and non-Gaussian noise in wheeled INS. The particle filter (PF) is an approximate Bayesian filtering algorithm relying on the Monte Carlo (MC) principle. Due to their ability to deal with non-linearities and non-Gaussian noise, the PF has become very attractive for navigation applications [33,34]. It constructs a point mass representation of a state vector using a large number of random samples, referred to as particles, to explore the state space. By modifying the weights and positions of particles, PF aims to approximate the probability distribution of random variables and obtain the minimum variance estimation [35]. Although a large number of particles improves the accuracy of state vector estimation, they also heavily increase the computational burden [36].

In this paper, we adopt the concept of PF to deal with non-linearities and non-Gaussian noise. To ensure a high level of estimation accuracy and limit the number of particles, we proposed a hybrid EKF-PF (referred as EPF) to continuously estimate the land vehicle navigation states, in which the latest observation is incorporated into PF to reduce the variance of particles with the approximation of the state through local linearization. In other words, the proposal distribution is generated by the EKF.

The implementation details of the EPF is given as follows:

Step 1: Initialization

1. Initialize the system states and their covariance matrix, randomly generate independent and identically distributed N samples ${\left\{x_{0}^{i}\right\}}_{i=1}^{N}$ from the prior probability distribution $p(x_{0})$.
2. Assign an equal weight of 1/N to all particles ${\left\{x_{0}^{i}\right\}}_{i=1}^{N}$.

Step 2: Time Propagation

1. When receiving the IMU measurements, calculate the PVA states for each particle through the INS mechanization algorithm.
2. For each particle, calculate the transition matrix based on the error model in Equations (5) and (6) and predict the covariance matrix of the system states using the following equation:

(33) $$P_{k|k-1}^{i}=\Phi_{k}^{i}P_{k-1|k-1}^{i}{\left(\Phi_{k}^{i}\right)}^{T}+Q_{k}$$

where P is the variance matrix, Φ is the transition matrix, Q is the processing noise of the system mode, and k is the time epoch.

Step 3: Proposal Distribution Generation

1. When the observation is available, implement the EKF update for each particle based on Equations (34)–(36).

(34) $$K_{k}^{\left(i\right)}=P_{k|k-1}^{\left(i\right)}{\left(H_{k}^{\left(i\right)}\right)}^{T}{\left[H_{k}^{\left(i\right)}P_{k|k-1}^{\left(i\right)}{\left(H_{k}^{\left(i\right)}\right)}^{T}+R_{k}\right]}^{-1}$$

(35) $${\hat{x}}_{k}^{i}=x_{k|k-1}^{i}+K_{k}^{i}\left(z_{k}-{\hat{z}}_{k}^{i}\right)$$

(36) $$P_{k}^{i}=\left(I-K_{k}^{i}H_{k}^{i}\right)P_{k|k-1}^{i}{\left(I-K_{k}^{i}H_{k}^{i}\right)}^{T}+K_{k}^{i}R_{k}{\left(K_{k}^{i}\right)}^{T}$$

where H is the design matrix of the measurement model; R is the measurement noise; K is the Kalman gain; z is the observation; and k is the time epoch.

2. Sample a new state from the Gaussian proposal distribution based on Equation (37).

(37) $$x_{k}^{i}∼q(x_{k}|x_{k-1}^{i},z_{k})=N({\hat{x}}_{k}^{i},P_{k}^{i})$$

3. For i=1…N, evaluate the importance weights of each particle according to Equation (38).

(38) $$w_{k}^{i}=w_{k-1}^{i}p(z_{k}\left|x_{k}^{i}\right.)$$

4. Normalize the weight for each particle, as stated in Equation (39).

(39) $$w_{k}^{i*}=w_{k}^{i}/\sum_{j=1}^{N}w_{k}^{j}$$

Step 4: Resampling

1. Compute the effective sample size and threshold based on Equation (40)

(40) $$N_{eff}=1/\sum_{i=1}^{N}{\left(w_{k}^{i}\right)}^{2},\;N_{th}=2N/3$$

where $N_{eff}$ is the computed effective sample size, and $N_{th}$ is the threshold value.

2. If $N_{eff}>N_{th}$, the particles remain as such, i.e., $x_{k}^{i*}=x_{k}^{i}$ for each particle with weight $w_{k}^{i*}$; otherwise, implement the resampling.

3. Construct the cumulative distribution function according to Equation (41), and generate a systematic sampling point according to Equation (42). For each $u_{j}$, find the index i, satisfying $c_{i-1}<u_{j}\leq c_{i}$. Eventually, copy the particles and reset the weights according to Equations (43) and (44).

(41) $$c_{0}=0,\;c_{i}=\sum_{j=1}^{i}w^{j},\;i=1,\ldots ,N$$

(42) $$u_{0}∼U[0,1/N],\;u_{j}=u_{0}+\frac{j-1}{N},\;j=1,\ldots ,N$$

(43) $$x_{k}^{j\_new}=x_{k}^{i},\;P_{k}^{j\_new}=P_{k}^{\left(i\right)}$$

(44) $$w_{k}^{j}=\frac{1}{N},\;j=1,\ldots ,N$$

Step 5: State Estimation

1. The state estimation and its corresponding covariance matrix are calculated using all particles, based on Equation (45),

(45) $${\hat{x}}_{k}=\sum_{i=1}^{N}w_{k}^{i}x_{k}^{i},{\hat{P}}_{k}=\sum_{i=1}^{N}w_{k}^{i}(x_{k}^{i}-{\hat{x}}_{k}){\left(x_{k}^{i}-{\hat{x}}_{k}\right)}^{T}$$

## 5. Kinematic Field Test Results and Analysis

This section presents the results of the kinematic field test to verify the observability characteristics of wheeled INS and investigate its positioning performance. Firstly, the field test equipment setup is introduced with the test trajectories; secondly, the covariance analysis is present to verify the system observability; thirdly, the PVA errors of wheeled INS with EKF are investigated with a comparison to the OD/NHC/INS; moreover, EPFs with different particle numbers are also investigated to find a compromise between estimation accuracy and computational complexity.

### 5.1. Experimental Setup

**Figure 3:**
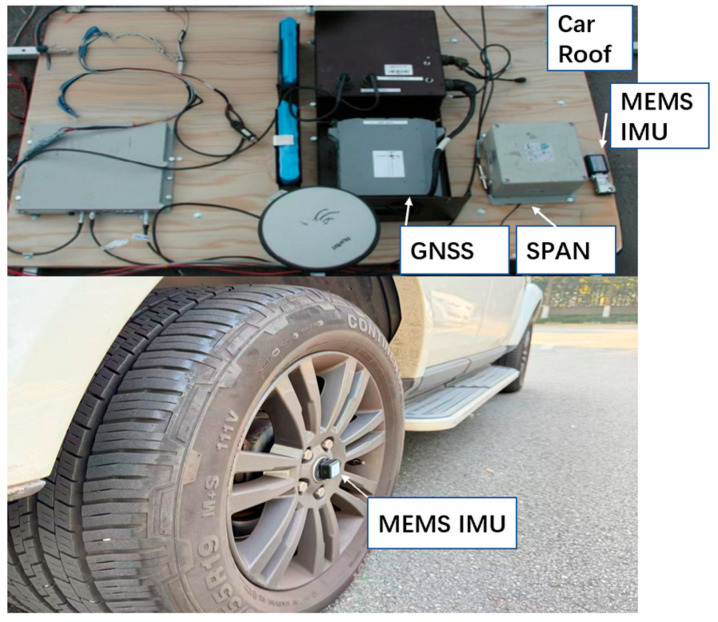
Equipment set-up for the field test.

The kinematic field tests were conducted based on a land vehicle, and the employed equipment is shown in Figure 3. A MEMS IMU, which includes a 9-axis inertial module (H30), a SD card to collect data, and a Bluetooth module for data transmission, was mounted on the wheel center. An equipment rack was mounted onto the vehicle’s roof, in which a SPAN system (including a GNSS receiver, an antenna, and a high-end IMU) was installed to provide the ground truth. The traditional OD/NHC/INS was also implemented to provide a comparison to the wheeled INS, and an identical MEMS IMU was mounted on the equipment rack. The odometer data was read from the vehicle’s built-in sensor through the OBD II interface using a device, namely a CarChip Pro. According to the manufacturer’s datasheet, the precision of the forward velocity data is 0.1 m/s. Table 2 lists the error parameters for the used MEMS IMUs and the high-end IMU in the SPAN system.

The reference solution was generated through a precise point positioning (PPP)/INS integrated method [37]. Both wheeled INS and OD/NHC/INS are dead-reckoning algorithms, and their initial attitude and position were obtained directly from the reference solution. The displacement and attitude misalignment between the w-frame and the s-frame in wheeled INS was calibrated and compensated in advance. An initial estimate of the gyro biases was obtained using the static IMU data [38].

**Figure 4:**
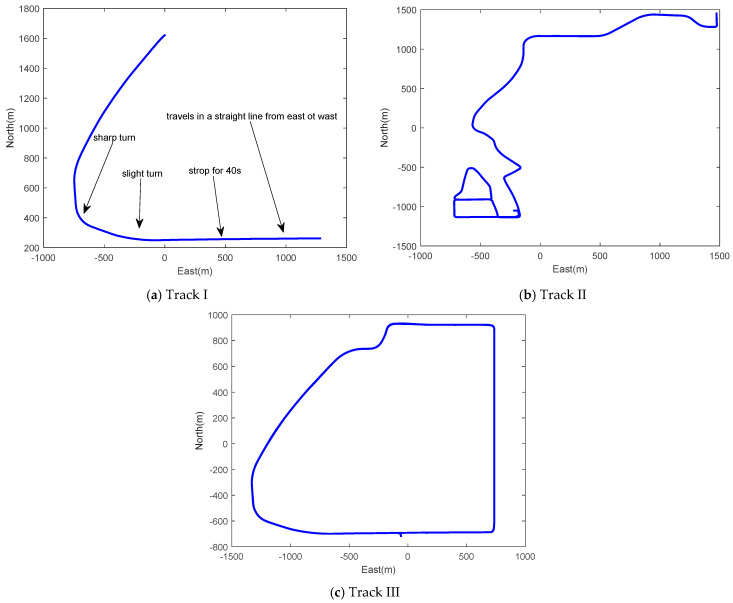
Test trajectories.

Three test trajectories are employed for the field test as shown in Figure 4. Track I is a short trajectory with a total length of over 3500 m, including driving in a straight-line, turning motion, and stops, and it is used to verify the observability of wheeled INS during linear and turning motions. Track II is a one-way trajectory with a wide range of vehicle dynamics, and Track IV is a large-loop trajectory, which the vehicle traveled around four times. Both tracks are regular urban roads, and there are a few bumpy road sections and uphill and downhill sections (the inclined angle is about 5–10°). Both Tracks II and III are employed to evaluate the positioning performance of the proposed navigation strategy. Vehicle dynamics information for the three trajectories is summarized in Table 3.

### 5.2. Observability Verification of Wheeled INS

The observability Gramian matrix was also employed to investigate the degree of observability for different error states. Table 4 summarizes the eigenvalues and eigenvectors of the Gramian matrix in different dynamic conditions.

A greater eigenvalue indicates higher observability, and the eigenvector gives the corresponding error state or its linear combinations. The obtained observability/Gramian results are consistent with the previous observability analysis presented in Section 3, and we found the following information regarding observability.

1. Under uniform linear motion, the observability of the 17-dimensional state vector can be divided into five levels based on the eigenvalues. The highest observability is for the velocity errors for the upward and eastward directions, which can be directly observed from the measurements (since the vehicle moves from east to west, the heading error has no effect on the eastward velocity). The second level is the attitude error for the northward direction, which can be derived from the time rate of the velocity measurements. The third level includes the Y-axis and Z-axis accelerometer errors and gyroscope errors. Due to the absence of acceleration, the displacement, gyroscope scale factor errors, and non-orthogonal errors are coupled with the accelerometer and gyroscope biases, forming jointly observable states. The fourth level is the coupled joint state of the attitude error of the eastward direction and the X-axis accelerometer error. The last level includes the velocity error for the northward direction and the azimuth error. This is because the azimuth error is hardly observable, and its error projects onto the northward velocity error.
2. Acceleration primarily enhances the observability of the Y-axis and Z-axis accelerometer biases, gyroscope biases, as well as the displacement, scale factor errors, and non-orthogonal errors. Based on eigenvalues, the observability of all error states can be divided into four levels. The highest observability remains with the velocity errors of the upward and eastward directions. The second level includes the attitude error for the northward direction, Y-axis and Z-axis accelerometer biases, and gyroscope biases, as well as the displacement, scale factor errors, and non-orthogonal errors. The third level is the coupled joint state of the attitude error for the eastward direction and the X-axis accelerometer error. The last level remains the velocity error of northward direction and the azimuth error.
3. Circular motion enhances the observability of the attitude error for the east direction and the X-axis accelerometer bias but simultaneously reduces the observability of the velocity error for the eastward direction. This is because circular motion projects the azimuth error onto the eastward velocity. Based on eigenvalues, the observability of all states can be divided into three levels. The first level includes the directly observable vertical velocity error. The second level includes the horizontal attitude errors, accelerometer and gyroscope biases, displacement, gyroscope scale factor errors, and non-orthogonal errors. The third level includes the hardly observable azimuth error, as well as the horizontal velocity errors affected by it.

It is notable that the inertial navigation error model assumes that “true state = estimated state + error state” and is developed through linearization around the estimated state trajectory. Therefore, the analysis only addresses the observability of the error states, and it is only valid when the errors are insignificant.

Covariance analysis is usually adopted to quantify the system observability by monitoring the decrease in the error state covariance matrix [39,40]. If the variance of a state diverges in time or tends to remain unchanged, its observability is considered to be low or unobservable. In contrast, if the variance of an error state decreases, the faster the reduction, the higher its observability.

**Figure 5:**
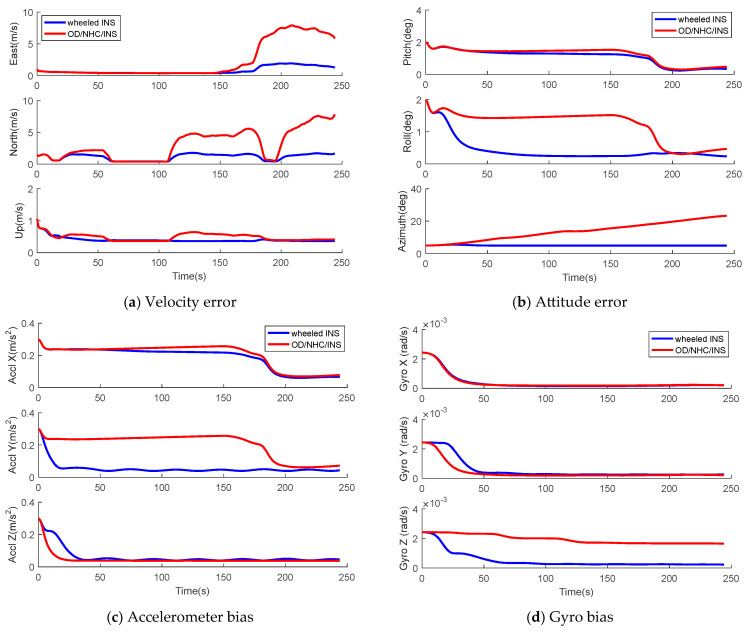
STDs of PVA error and sensor error states in wheeled INS and OD/NHC/INS for Track I.

**Figure 6:**
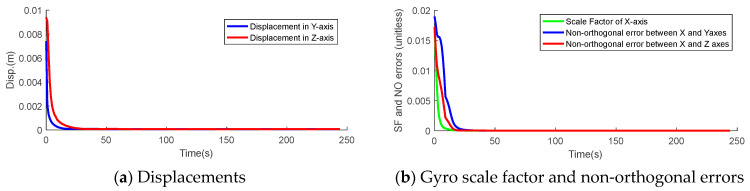
STDs of the displacements, gyro scale factor, and non-orthogonal errors.

As shown in Figure 4a, the vehicle first travels in a straight line from east to west for about 150 s, and stops for about 40 s. Then, the vehicle makes a slight turn and sharp turn afterwards at 150 s and 170 s, consecutively. Figure 5 and Figure 6 illustrate the STD of all states extracted from the covariance matrix of both wheeled INS and OD/NHC/INS for Track I. We obtained the following findings for wheeled INS, which are consistent to the previous analysis in Section 3:

1. The variance in the velocity error in the upward direction, the accelerometer biases of Y- and Z-axes, and the gyro biases of the X-, Y- and Z-axes, as well as the displacements between the IMU center to the wheel rotation center and the scale factor and non-orthogonal error states, are quickly reduced, which indicates that these error states are observable.
2. During the initial 150 s, as the vehicle is traveling along the east–west direction, the pitch error $\varepsilon_{E}$ and roll error $\varepsilon_{N}$ become $\varepsilon_{Y}$ and $\varepsilon_{X}$, respectively, according to Equation (32); therefore, the variance in roll errors is quickly reduced. As $\varepsilon_{Y}$ and the accelerometer bias of the X-axis $\gamma_{X}$ are coupled error states and only their linear combination is observable, the variance in both error states almost remains unchanged until the vehicle’s sharp turn makes them become observable after 170 s.
3. As the azimuth error is unobservable, its variance almost remains unchanged, which causes the unreduced variance in the velocity errors for the eastward and northward directions. There are two periods during which the projections of azimuth error variance in velocity errors are eliminated. The first period is the initial 150 s, during which the vehicle travels from east to west and the velocity error of the eastward direction $\delta v_{E}$ becomes $\delta v_{Y}$. The second period is the vehicle’s static period from about 60–100 s, during which the velocity error in the north direction $\delta v_{N}$ becomes $\delta v_{X}$, according to Equation (31).

Compared to the wheeled INS, we can verify that the deficiencies in OD/NHC/INS are as follows:

1. The gyro bias of the Z-axis is unobservable and its variance remains almost unchanged, which leads to the variance in another unobservable bias, azimuth error, diverging over time. This leads to a much greater variance in the velocity error for the eastward and northward directions compared to the wheeled INS.
2. The accelerometer bias of Y-axis and the roll errors are coupled states, and they cannot be separately observed during the linear motion, which also affects the observability of velocity errors in the upward direction according to Equation (31), although they become observable with sharp turning motions.

The initial bounds for the displacements are set to 1 cm in stochastic filtering, while they are set to 1 × 10^−2^ for the gyro scale factor and the non-orthogonal errors. According to the observability analysis in Section 3, forward accelerations are required to separate these error states from the accelerometer and gyro errors and improve their observability. As shown in Figure 6, the STDs of the displacements and gyro scale factor, as well as non-orthogonal errors, converge quickly with acceleration. The STD of the displacement in the Y-axis converges slightly faster than that in the *Z*-axis due to its higher observability, according to Table 4. Similarly, the STDs of the gyro scale factor of the rotation axis also converges faster than non-orthogonal errors.

Observability analysis indicates that the accelerometer in the vertical direction and the gyroscope in the horizontal direction are independently observable in an OD/INS. However, the observability of accelerometers in the horizontal direction and the gyroscope along the azimuth axis is poor. The fundamental principle by which IMU rotation enhances the observability of sensor errors lies in reorienting the originally horizontal accelerometer towards the vertical direction and the originally vertical gyroscope towards the horizontal direction, thereby making them observable. Therefore, in wheeled INS, while the observability of the Y-axis accelerometer and the Z-axis gyroscope is improved, the convergence speed of the Z-axis accelerometer and the Y-axis gyroscope is slower, as they are rotated towards other orientations.

### 5.3. Navigation Performance of Wheeled INS

#### 5.3.1. Comparison Between Wheeled INS and OD/NHC/INS

**Figure 7:**
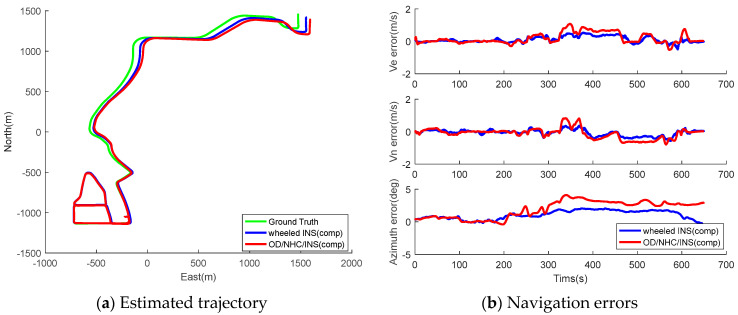
Estimated trajectories and navigation errors of wheeled INS and OD/NHC/INS with compensation for initial gyro biases for Track II.

**Figure 8:**
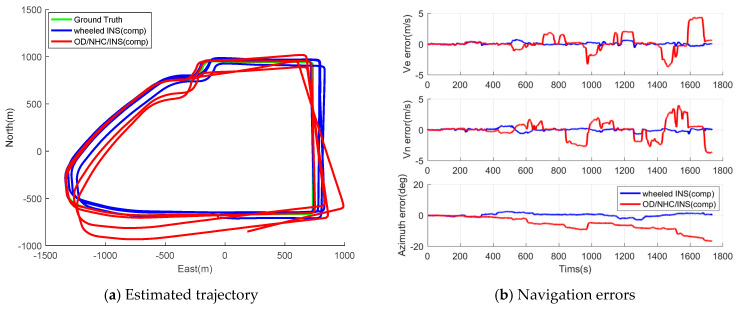
Estimated trajectories and navigation errors of wheeled INS and OD/NHC/INS with compensation for initial gyro biases for Track III.

The collected data of both wheeled INS and OD/NHC/INS are processed with gyro biases compensated using initial estimates from the static IMU data, and the EKF is first employed to estimate the inertial errors. For the land vehicle navigation, we mainly focus on the position performance of the horizontal plane. Figure 7 and Figure 8 illustrate the estimated trajectory and navigation errors of both wheeled INS and OD/NHC/INS for Tracks II and III. The maximum and RMS of the horizontal position, velocity, and azimuth errors for both tracks are summarized in Table 5. In addition to the statistics for PVA errors, we also adopted a different metric to evaluate the positioning performance of wheeled INS, which has been used in other studies [22,41]. The trajectory is segmented by a certain distance, increment l (50 m), from the initial point; then, the maximum horizontal position error drift is calculated for l, 2l, 31, etc. Finally, the mean value (MEAN) and standard deviation (STD, 1σ) of these segmented drift rates are computed as indicators of positioning performance [21].

Based on the figures and navigation error statistics, we can obtain the following information:

1. The proposed wheeled INS is able to offer reliable navigation solutions in GNSS-denied environments. The heading error and maximum position drift rate is 1.28° and 0.85% for Track II, while the same metric for Track III is 1.42 and 0.54%. The maximum position drift for Track III is even lower than that of the Track II. This is because the position errors can be suppressed to some extent due to loops in the trajectory, as the position error usually drifts along one direction in INSs.
2. The wheeled INS outperforms the OD/NHC/INS in terms of positioning accuracy overall; the RMS of horizontal position, velocity errors, and azimuth errors—as well as the maximum position drift rate of wheeled INS—are improved by 30.94%, 36.00%, 44.83%, and 12.37%, respectively, compared to the OD/NHC/INS for Track II. The same figures for wheeled INS are improved by 56.06%, 76.29%, 81.56%, and 46.53%, respectively, for Track III. As Track III is a much longer trajectory than Track II, this indirectly reflects the fact that the navigation errors can be well mitigated in wheeled INS, and the longer the distance traveled, the greater improvements over OD/NHC/INS can be observed.
3. During the initial period, the wheeled INS solutions are close to those of the OD/NHC/INS. This is because the gyro bias of the Z-axis remains stable and close to its initial estimates, which only introduces small azimuth and velocity errors in both systems.
4. After the initial periods, the gyro bias of the Z-axis drifts away from its initial estimates, and the compensation for these errors depends on its observability in both systems. In wheeled INS, the gyro bias can be well estimated due to its high observability, which effectively limits the azimuth and horizontal velocity errors; in contrast, such error is unobservable and hardly to be estimated in OD/NHC/INS, which introduces accumulated azimuth and horizontal velocity errors.

**Figure 9:**
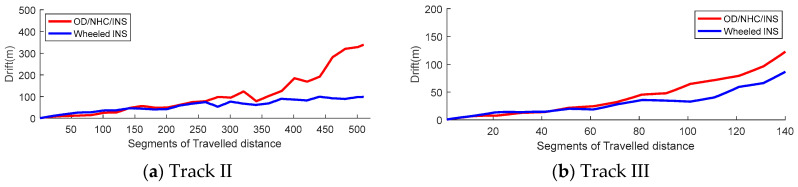
Segment-based drift.

Figure 9 illustrates the segment-based drift for both tracks. Apparently, the OD/NHC/INS drifts much faster than wheeled INS in both tracks.

**Figure 10:**
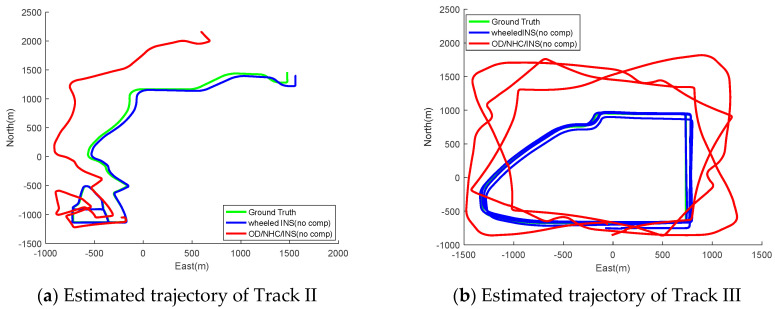
Estimated trajectories of wheeled INS and OD/NHC/INS without compensation for initial gyro biases for Tracks II and III.

As the gyro bias drifts fast for most consumer-grade MEMS IMUs, their online estimation and compensation are crucial for an integrated navigation system. The deficiency of the OD/NHC/INS in terms of system observability makes it very sensitive to the gyro bias compensation before each run. Conversely, the improved system observability leads to the wheeled INS being immune to gyro bias compensation in advance, which is more suitable for applications when initial estimates of gyro biases are difficult to obtain. Figure 10 illustrates the estimated trajectories of both systems for both Tracks II and III without compensation for gyro biases. Thanks to the online estimation of gyro bias perpendicular to rotation axes, the estimated trajectory of wheeled INS is still consistent with the ground truth, while the navigation solution drifted far away in OD/NHC/INS due to the failed online estimation of gyro biases in the *Z*-axis. The navigation error statistics for both systems without compensation for initial gyro biases are summarized in Table 6. The RMS position errors drift over thousands of meters in OD/INS/NHC without compensation for initial gyro biases; conversely, the positioning metrics of wheeled INS are very close to those with compensation.

#### 5.3.2. Positioning Performance of Wheeled INS with EPF and EKF

**Figure 11:**
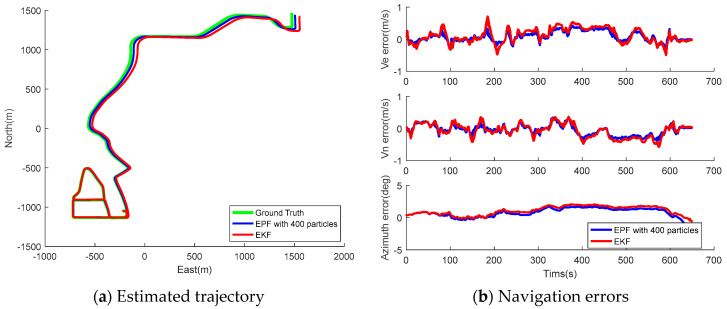
Estimated trajectory and navigation errors of EPF with 400 particles for Track II.

**Figure 12:**
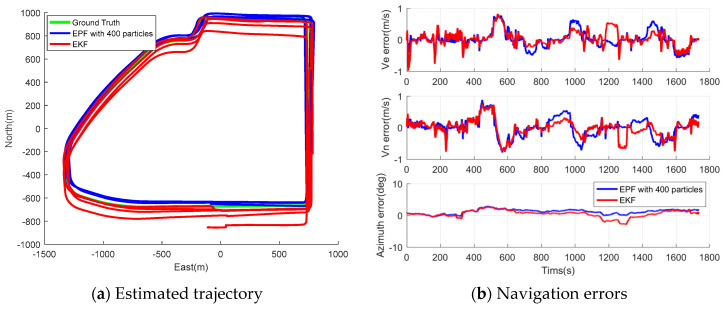
Estimated trajectory and navigation errors of EPF with 400 particles for Track III.

The collected wheel-mounted data was processed using the proposed EPF to investigate its ability to estimate the vehicle navigation states. Moreover, the impact of initial particle numbers on the EPF estimation accuracy was also studied, and the tested particle number ranged from 100 to 600. Figure 11 and Figure 12 present the estimated trajectory and navigation errors using EPF with 400 initial particles and EKF for Tracks II and III. Apparently, the EPF solution is more consistent to the ground truth, and the time sequence of its PVA errors is more stable compared to that of the EKF solution. Table 7 and Table 8 summarize the statistics for the navigation errors and execution time of EPF with a particle number ranging from 100 to 600 for Tracks II and III, respectively. The statistics for the same figures obtained from the EKF are also provided for comparison. We can obtain the following findings:

1. Generally speaking, the estimation errors reduced as the particle number increased, along with the execution time; however, at a certain point (a particle number of 400), the reduction rate of navigation error decreased, but the calculation time still increased rapidly; therefore, 400 was chosen as the suitable particle number to implement the EPF in wheeled INS.
2. The EPF outperforms the EKF in navigation solution accuracy overall; compared to the results of EKF, the RMS of horizontal position error, velocity errors, azimuth errors, and position drift rate of EPF with 400 particles are improved by 29.78%, 25.00%, 19.53%, and 29.41%, respectively, for Track II; the same figures of EPF with 400 particles are improved by 30.70%, 21.74%, 20.42%, and 12.96%, respectively, for Track III.
3. Finally, the proposed wheeled INS with EPF is able to provide superior autonomous navigation solution. In the tests, the heading error and maximum position drift rate were 1.01–1.3° and 0.47–0.60%, respectively.

## 6. Conclusions and Future Work

In this paper, an autonomous navigation system is proposed and implemented based on a single wheel-mounted MEMS IMU, namely the wheeled INS. To eliminate the detrimental centripetal accelerations and gyro error introduced by wheel rotation, the displacement between the IMU sensitive axes center and the IMU rotation center, as well as the gyro scale factor and non-orthogonal errors, are augmented to the state space and estimated online.

A comprehensive observability analysis is conducted for the proposed system, which established a theoretical foundation for the error characteristics of wheeled INS. With the wheel rotation, the improved system observability of wheeled INS leads to its superior navigation performance over the OD/NHC/INS. According to the kinematic field test results, the maximum position error drift of wheeled INS is only 0.54% with over 26 km traveled during the 1735 s test period. This represents an improvement of 46.53% compared to the drift in OD/NHC/INS.

The EPF is proposed to update the vehicle navigation states due to its ability to (1) deal with system non-linearity and non-Gaussian noise, and (2) simultaneously achieve a high level of estimation accuracy and tolerable computation complexity. With 400 particles, the maximum position drift obtained in EPF further improved to 0.47%. The obtained results are very promising, as only one single MEMS IMU is employed in the proposed autonomous navigation system.

Although the wheeled INS outperforms the OD/NHC/INS, it is not a completely observable system, and the azimuth error is barely observable during normal vehicle dynamics. As a result, it causes two potential problems: (1) the initial azimuth error cannot be corrected, and (2) the azimuth solution will eventually diverge without external heading updates. Future studies may consider employing the absolute heading from external aiding information, such as map matching, to make the system completely observable and further enhance the navigation performance.

## Figures and Tables

**Table 1:** Comparison of our study with the existing literature.

	Our Study	Prior Wheel-Mounted IMU	OD/NHC/INS
Observability analysis	Analyzed based on both observability matrix and Gramian results	Has not yet been conducted	Analyzed through covariance matrix
Stochastic filtering	EPF	EKF	EKF
Calibration of displacements	Online calibration	Manual calibration	Not applicable

**Table 2:** Error parameters of the MEMS IMU and SPAN.

	Accl *. Bias Stability (mg)	VRW * (m/s/√h)	Gyro Bias Stability (°/h)	ARW * (°/√h)
MEMS IMU (H30)	0.35	0.5	4	0.3
SPAN	0.075	0.06	0.45	0.06

* Accl. denotes the accelerometer, VRW denotes the velocity random walk, and ARW denotes the angular random walk.

**Table 3:** The vehicle dynamic information for three trajectories.

	Time Duration (s)	Total Distance (m)	Maximum Velocity (m/s)	Average Velocity (m/s)
Track I	246	3552	27.78	14.44
Track II	649	7262	18.47	11.19
Track III	1735	26312	28.85	15.17

**Table 4:** The eigenvalues and eigenvectors of the Gramian matrix.

	Uniform Linear Motion		Linear Motion with Acceleration		Circular Motion	
	Eigenvalue	Eigenvector	Eigenvalue	Eigenvector	Eigenvalue	Eigenvector
λ_1_	169.26	$\left\{\delta v_{E}\right\}$	172.62	{$\delta v_{E}$}	172.63	{$\delta v_{U}$}
λ_2_	168.01	$\left\{\delta v_{U}\right\}$	171.99	{$\delta v_{U}$}	90.19	{$\varepsilon_{N}$}
λ_3_	78.93	{$\varepsilon_{N}$}	88.05	{$\varepsilon_{N}$}	90.05	{$\varepsilon_{E}$}
λ_4_	9.97	{$\gamma_{Y}$,$l_{Y}$}	87.98	{$\gamma_{Y}$}	90.01	{$\gamma_{X}$}
λ_5_	9.21	{$\gamma_{Y}$,$l_{Y}$}	87.32	{$\gamma_{Z}$}	89.91	{$\gamma_{Y}$}
λ_6_	9.18	{$\gamma_{Z}$,$l_{Z}$}	86.11	{$l_{Y}$}	89.87	{$\gamma_{Z}$}
λ_7_	9.09	{$\gamma_{Z}$,$l_{Z}$}	84.60	{$l_{Z}$}	88.59	{$l_{Y}$}
λ_8_	3.72	{$d_{X}$,$\zeta_{X}$}	42.71	{$d_{X}$}	87.03	{$l_{Z}$}
λ_9_	3.66	{$d_{Z}$,$\zeta_{XZ}$}	41.50	{$d_{Z}$}	57.53	{$d_{X}$}
λ_10_	3.10	{$d_{Y}$,$\zeta_{XY}$}	40.18	{$d_{Y}$}	55.45	{$d_{Z}$}
λ_11_	3.01	{$d_{X}$,$\zeta_{X}$}	40.09	{$\zeta_{X}$}	54.24	{$d_{Y}$}
λ_12_	2.97	{$d_{Z}$,$\zeta_{XZ}$}	39.76	{$\zeta_{XZ}$}	53.14	{$\zeta_{X}$}
λ_13_	2.72	{$d_{Y}$,$\zeta_{XY}$}	39.34	{$\zeta_{XY}$}	52.59	{$\zeta_{XZ}$}
λ_14_	0.86	{$\varepsilon_{E}$,$\delta f_{X}$}	1.02	{$\varepsilon_{E}$,$\delta f_{X}$}	52.17	{$\zeta_{XY}$}
λ_15_	0.81	{$\varepsilon_{E}$,$\delta f_{X}$}	0.95	{$\varepsilon_{E}$,$\delta f_{X}$}	0.22	{$v_{E}$}
λ_16_	0.10	{$v_{N}$}	0.12	{$v_{N}$}	0.22	{$v_{N}$}
λ_17_	0.09	{$\varepsilon_{U}$}	0.10	{$\varepsilon_{U}$}	0.12	{$\varepsilon_{U}$}

**Table 5:** Navigation error statistics of wheeled INS and OD/NHC/INS with compensation for initial gyro biases for Tracks II and III.

Positioning Metrics		Horizontal Position Error (m)		Horizontal Velocity Error (m/s)		Azimuth Error (°)		Position Drift Rate (%)	
		Max	Rms	Max	Rms	Max	Rms	STD	Mean
Track II	Wheeled INS with comp.	85.10	48.06	0.71	0.32	2.11	1.28	0.35	0.85
	OD/NHC/INS with comp.	130.92	69.59	1.22	0.50	4.13	2.32	0.66	0.97
Track III	Wheeled INS with comp.	100.49	62.41	1.05	0.46	2.93	1.42	0.17	0.54
	OD/NHC/INS with comp.	337.81	142.04	4.47	1.94	17.48	7.70	0.70	1.01

**Table 6:** Navigation error statistics of wheeled INS and OD/NHC/INS without compensation for initial gyro biases for Tracks II and III.

Error Statistics		Horizontal Position Error (m)		Horizontal Velocity Error (m/s)		Azimuth Error (°)		Position Drift Rate (%)	
		Max	Rms	Max	Rms	Max	Rms	STD	Mean
Track II	Wheeled INS without comp.	99.34	53.71	0.88	0.40	2.58	1.56	0.51	0.90
	OD/NHC/INS without comp.	1051.12	531.82	7.83	4.01	33.47	20.16	2.87	11.44
Track III	Wheeled INS without comp.	104.77	64.94	1.11	0.57	3.29	1.71	0.38	0.60
	OD/NHC/INS without comp.	3042.24	1532.702	53.01	22.48	179.99	103.99	6.71	20.41

**Table 7:** Navigation error statistics of EPF with different particle numbers for Track II.

Positioning Metrics	Horizontal Position Error		Horizontal Velocity Error		Azimuth Error		Position Drift Rate		Execution Time
	Max	Rms	Max	Rms	Max	Rms	STD	Mean	(s)
EPF with 100 particles	82.12	46.12	0.63	0.31	2.08	1.26	0.34	0.81	35.80
EPF with 200 particles	72.39	40.44	0.51	0.28	1.96	1.16	0.31	0.71	37.06
EPF with 300 particles	64.43	35.89	0.47	0.25	1.82	1.07	0.28	0.64	44.42
EPF with 400 particles	60.70	33.75	0.44	0.24	1.81	1.03	0.26	0.60	57.90
EPF with 500 particles	59.51	33.07	0.43	0.24	1.81	1.01	0.25	0.60	77.51
EPF with 600 particles	58.97	32.76	0.43	0.24	1.81	1.01	0.25	0.59	103.22
EKF	85.10	48.06	0.71	0.32	2.11	1.28	0.35	0.85	13.77

**Table 8:** Navigation error statistics of EPF with different particle numbers for Track III.

Positioning Metrics	Horizontal Position Error		Horizontal Velocity Error		Azimuth Error		Position Drift Rate		Execution Time
	Max	Rms	Max	Rms	Max	Rms	STD	Mean	(s)
EPF with 100 particles	95.47	61.60	0.94	0.44	2.93	1.39	0.17	0.54	139.51
EPF with 200 particles	76.66	51.56	0.84	0.39	2.88	1.24	0.17	0.52	144.50
EPF with 300 particles	71.27	45.59	0.75	0.37	2.86	1.16	0.17	0.49	176.35
EPF with 400 particles	68.23	43.25	0.71	0.36	2.85	1.13	0.16	0.47	226.39
EPF with 500 particles	67.12	42.56	0.70	0.36	2.85	1.13	0.16	0.47	306.16
EPF with 600 particles	66.65	42.30	0.70	0.36	2.85	1.13	0.16	0.47	411.85
EKF	100.49	62.41	1.05	0.46	2.93	1.42	0.17	0.54	53.31

## Data Availability

Data can be requested from the corresponding author.

## Appendix A

### Observability Analysis for OD/NHC/INS

The observability of OD/NHC/INS is first analyzed under uniform linear motion; then, the impact of forward accelerations and turning motion on system observability are given. As IMU is relatively static to the vehicle, the displacement states, gyro scale factor, and non-orthogonal errors are excluded from the state space. The first time derivative of the accelerometer and gyro biases is zero in the absence of noise, as they are modeled as the random walk. The time derivatives of the observations during the uniform linear motion are given in Equation (A1).

(A1) $$\left[\begin{array}{c} \delta {\dot{v}}^{b}\\ \delta {\ddot{v}}^{b}\\ \delta {\dddot{v}}^{b}\\ \vdots \\ \overset{\left(n\right)}{\delta v^{b}} \end{array}\right]=\left[\begin{array}{cccc} 0_{3\times 3} & F & I_{3\times 3} & 0_{3\times 3}\\ 0_{3\times 3} & 0_{3\times 3} & 0_{3\times 3} & F\\ 0_{3\times 3} & 0_{3\times 3} & 0_{3\times 3} & 0_{3\times 3}\\ \vdots & \vdots & \vdots & \vdots \\ 0_{3\times 3} & 0_{3\times 3} & 0_{3\times 3} & 0_{3\times 3} \end{array}\right]\left[\begin{array}{c} \delta v^{b}\\ \varepsilon^{b}\\ \gamma^{b}\\ d^{b} \end{array}\right]$$

This shows that the third-order and above time derivatives of observations become null space. From the observation, as well as its first- and second-order time derivatives, the observable states or linear combination of states are $\delta v_{x},\delta v_{y},\delta v_{z}$. $-g\varepsilon_{Y}+\gamma_{X}$, $g\varepsilon_{X}+\gamma_{Y}$, $\gamma_{Z}$, $d_{X}$, and $d_{Y}$; the attitude error $\varepsilon_{Z}$ and the gyro bias of the Z-axis $d_{Z}$ are unobservable.

In the presence of forward accelerations, the first- and second-order observable time derivatives can be re-calculated as $\delta {\dot{v}}^{b}=\left[\begin{array}{c} -g\varepsilon_{Y}+f_{Y}\varepsilon_{Z}+\gamma_{X}\\ g\varepsilon_{X}+\gamma_{Y}\\ -f_{Y}\varepsilon_{X}+\gamma_{Z} \end{array}\right]$ and $\delta {\ddot{v}}^{b}=\left[\begin{array}{c} -gd_{Y}+f_{Y}d_{Z}\\ gd_{X}\\ -f_{Y}d_{X} \end{array}\right]$, respectively. Although the forward acceleration makes the error states $\varepsilon_{X}$, $\gamma_{Y}$, and $d_{Z}$ become theoretically observable, it barely affects their observability in practice, as it is much smaller than the gravity on the vehicle under normal driving conditions.

During the turning motion, the time derivatives of the velocity observation are given in Equation (A2). Then, the differential equation $\delta v_{n,n-1}=\overset{\left(n\right)}{\delta v^{b}}-\Omega_{Z}\overset{\left(n-1\right)}{\delta v^{b}}$ can be calculated as shown in Equation (A3). Apparently, the vehicle rotation rate matrix $\Omega_{Z}$ only affects the observability attitude error and gyro biases. As $\Omega_{Z}^{3}=-\omega_{Z}^{2}\Omega_{Z}$, the observability is only determined by $\delta v_{1,0}$, $\delta v_{2,1}$, and $\delta v_{3,2}$, which are given in Equations (27), (A4), and (A5), respectively.

(A2) $$\left[\begin{array}{c} \delta {\dot{v}}^{b}\\ \delta {\ddot{v}}^{b}\\ \delta {\dddot{v}}^{b}\\ \vdots \\ \overset{\left(n\right)}{\delta v^{b}} \end{array}\right]=\left[\begin{array}{cccc} \Omega_{Z} & F & I_{3\times 3} & 0_{3\times 3}\\ \Omega_{Z}^{2} & \Omega_{Z}F+F\Omega_{Z} & \Omega_{Z} & F\\ \Omega_{Z}^{3} & \Omega_{Z}^{2}F+\Omega_{Z}F\Omega_{Z}+F\Omega_{Z}^{2} & \Omega_{Z}^{2} & \Omega_{Z}F+F\Omega_{Z}\\ \vdots & \vdots & \vdots & \vdots \\ \Omega_{Z}^{n} & \sum_{i=0}^{n-1}\Omega_{Z}^{n-1-i}F\Omega_{Z}^{i} & \Omega_{Z}^{n-1} & \sum_{i=0}^{n-2}\Omega_{Z}^{n-2-i}F\Omega_{Z}^{i} \end{array}\right]\left[\begin{array}{c} \delta v^{b}\\ \varepsilon^{b}\\ \gamma^{b}\\ d^{b} \end{array}\right]$$

(A3) $$\delta v_{n.n-1}=F\Omega_{Z}^{n-2}(\Omega_{Z}\varepsilon^{b}+d^{b})$$

(A4) $$\delta v_{21}=\left[\begin{array}{c} -g(\omega_{Z}\varepsilon_{X}+d_{Y})\\ -g(\omega_{Z}\varepsilon_{Y}-d_{X})-f_{X}d_{Z}\\ f_{X}(\omega_{Z}\varepsilon_{X}+d_{Y}) \end{array}\right]$$

(A5) $$\delta v_{3,2}=\left[\begin{array}{c} -g\omega_{Z}(-\omega_{Z}\varepsilon_{Y}+d_{X})\\ -g\omega_{Z}(\omega_{Z}\varepsilon_{X}+d_{Y})\\ f_{X}\omega_{Z}(-\omega_{Z}\varepsilon_{Y}+d_{X}) \end{array}\right]$$

From $\delta v_{2,1}$ and $\delta v_{3,2}$, the combination of error states $\omega_{Z}\varepsilon_{X}+d_{Y}$ and $\omega_{Z}\varepsilon_{Y}-d_{X}$ can be directly determined, from which the attitude errors $\varepsilon_{X}$ and $\varepsilon_{Y}$ can be derived. Then, their coupled error states, $\gamma_{X}$ and $\gamma_{Y}$, can also be derived from Equation (13). As the centripetal acceleration $f_{X}$ is much smaller than gravity, the attitude error $\varepsilon_{Z}$ and gyro bias $d_{Z}$ are still difficult to observe in the second element of $\delta v_{1,0}$ and $\delta v_{2,1}$. In summary, the observable states or combination of states are $\delta v_{x}$, $\delta v_{y}$, $\delta v_{z}$, $\varepsilon_{X}$, $\varepsilon_{Y}$, $\gamma_{X}$, $\gamma_{Y}$, $\gamma_{Z}$, $d_{X}$, and $d_{Y}$; the attitude error $\varepsilon_{Z}$ and the gyro bias of the *Z*-axis $d_{Z}$ are unobservable.
